# The associations between screen time and mental health in adolescents: a systematic review

**DOI:** 10.1186/s40359-023-01166-7

**Published:** 2023-04-20

**Authors:** Renata Maria Silva Santos, Camila Guimarães Mendes, Guilherme Yanq Sen Bressani, Samara de Alcantara Ventura, Yago Jean de Almeida Nogueira, Débora Marques de Miranda, Marco Aurélio Romano-Silva

**Affiliations:** 1 Graduate Program Molecular Medicine, Federal University of Minas Gerais (UFMG), Belo Horizonte, Brazil; 2 Graduate Program Children and Adolescent Health, Federal University of Minas Gerais (UFMG), Belo Horizonte, Brazil; 3 Scientific Research Program Molecular Medicine, Federal University of Minas Gerais (UFMG), Belo Horizonte, Brazil; 4 Department of Pediatrics, Federal University of Minas Gerais (UFMG), Belo Horizonte, Brazil; 5 Department of Mental Health, Federal University of Minas Gerais (UFMG), Belo Horizonte, Brazil

**Keywords:** Screen time, Mental health, Adolescents, Media

## Abstract

**Background:**

Adolescents have extensive use of screens and, they have common complains related to mental health. Here a systematic review was done to understand the association between screen time and adolescent’s mental health.

**Method:**

This review was conducted in compliance with Preferred Reporting Items for Systematic Reviews and Meta-Analyses – PRISMA. An update search was performed in January 2023 with the following keywords: “screen time,“ “adolescent,“ and “mental health” on PubMed, PsycINFO and Scopus databases.

**Results:**

50 articles were included, most have found associations between screen exposure and mental health in adolescents. The most used device by adolescents was the smartphone and the use on weekdays was associated with diminished mental well-being. Social media use was negatively associated with mental well-being and, in girls, associated at higher risk for depression.

**Conclusion:**

Excessive screen time in adolescents seems associated with mental health problems. Given the profusion and disparity of the results, additional studies are needed to clarify elements such as the screen content or the interaction of adolescents with different screen devices.

**Systematic review registration:**

PROSPERO CRD42022302817.

**Supplementary Information:**

The online version contains supplementary material available at 10.1186/s40359-023-01166-7.

## Introduction

Adolescence represents a phase of increased risk for the emergence of mental health problems [1, 2]. According to the 2021 update of the World Health Organization (WHO) [3], it is estimated that 14% of young people between 10 and 19 years old have mental health problems, which represents, globally, 13% of all diseases that affect this population. Depression, anxiety, and behavioral disorders are among the leading causes of illness and disability in adolescents, and suicide is already the fourth leading cause of death among 15–19-year-olds [3].

The current generation of teenagers is growing up immersed in a world saturated with electronic media, they did not know the times before the internet and for this reason, they are called “digital natives” [4]. Media use of screen-based electronic devices is extensive, including television (TV) programs watching and using computers, tablets, and smartphones. In the last 10 years, the use of mobile Internet devices has increased exponentially, becoming part of everyday life [5].

The increased use of screens has been noticeable and amplified in the COVID-19 pandemic due to isolation and restrictions on other leisure activities [6]. The American Academy of Pediatrics (AAP) recommends that children over age five through adolescence be exposed to less than 2 h of screen time (ST) per day. However, a large percentage of adolescents already exceed this recommendation [7]. In general, these media-related activities occupy about 6 to 9 h of a young American’s day, excluding housework and schoolwork [2, 4].

Adolescents are particularly susceptible to the opportunities and risks of new technologies [8]. The development of socio-affective brain circuits can increase sensitivity to social information, impulsiveness toward rewards, as well as a preoccupation with peer evaluation [2]. The growing suggestion that excessive screen time is related to recent increases in mental health problems among young people has been the focus of research [9–11]. However, reviews of this nature were either restricted to children [9] or included several age groups [10–12]. In addition, the specific focus on a given symptom [9, 12], or the concern with providing recommendations and strategies [10], reinforces the need for a more detailed investigation of this relationship among adolescents.

Results from previous reviews are mixed [13–16]. In the general population, research done during COVID-19 pandemic has found most evidence indicating negative effects of long screen time on mental health (MH) [10]. Among children, an association was found between screen time and internalizing and externalizing behavior problems [9]. In adolescents, the association between social media use and psychological well-being was negative but very small [17]. Together, adolescent children and young adults showed a small to very small association between screen time and depressive symptoms, varying between different devices and uses [11]. Furthermore, methodological issues such as cross-sectional design, sampling and measurements can weaken the evidence [17, 18].

Accumulated evidence indicates that screen time may be associated with aspects of the adolescent’s mental health. However, the direction of these associations is not yet clear, the literature still lacks comprehensive and detailed research. With this in mind, this review aims to contribute to the understanding of the effects that exposure to screens can promote on a wide range of the mental health aspects of adolescents, which were previously researched such as flourishing (synonymous with a high level of mental well-being), life satisfaction, self-efficacy, self -concept Physical, psychosocial difficulties, conduct problems, hyperactivity/inattention and pro-social behavior, symptoms of internalization and externalization, positive mental health, and mental well-being. In addition, this review included the various screen-based devices most used by this population. The goal was to raise evidence that could increase knowledge about the interactions that adolescents set with screens, which may have potential additive increase in exposure time and effect on the mental health of this population.

## Method

This review was conducted in compliance with Preferred Reporting Items for Systematic Reviews and Meta-Analyses – PRISMA [19] and was registered at PROSPERO, under number CRD42022302817. A search was performed on 12/27/2021 with the following question: Is there any association between screen time and mental health in adolescents? For this, the following keywords were used: “screen time,” “adolescent,” and “mental health,” combined with the operator AND on databases PubMed, PsycINFO, and Scopus. An additional search was performed to update the research on 01/18/2023, using the same strategy and the same databases, totaling six more studies included. Table 1 presents the search strategies (Additional file 1: Table 1). The adopted strategy tried to increase the sensitivity of the searches, expanding the range of outcomes with the umbrella term mental health, and searching in all or any fields of the articles. In accordance with the PICO strategy, the study population will be composed of individuals in the adolescence phase and the intervention will be represented by exposure to screens. As outcomes, any aspects related to mental health will be considered, including measures related to mental well-being, like life satisfaction, and the control groups may be absent from the observational studies included. With the volume of data captured, considering previous reviews, the addition of records from the reference lists of the included articles was not considered.

Inclusion criteria: (1) articles that directly assessed associations between screen time [i.e., the amount of time spent using a device with a screen such as a smartphone, computer or video game console (active screen, screens that allow motor interaction and responsivity), or television (TV) and videos (passives screens), for entertainment or educational use] and mental health, that is, (mood, internalization or externalization problems, sleep disorders, aspects related to mental well-being such as satisfaction with life, self-esteem and self-efficacy, among others), with at least one variable evaluated; (2) studies that measured mental health outcomes through validated scales/instruments; (3) studies with adolescents, average age between 12 and 18 years; (4) articles in English, published in the last 10 years. Exclusion criteria: (1) studies carried out with adolescents diagnosed with problematic internet use, (2) sample composed of adolescents already diagnosed with mental health problems or being followed up in mental health/psychiatry outpatient clinics, (3) research that used screen-based devices to aid functionality and (4) case reports and case series.

The screening procedure was performed in pairs, including an initial independent search. After duplicate records were deleted, the titles and abstracts of each study were screened according to the inclusion and exclusion criteria. The articles eligible for full reading were selected and the two authors discussed the results and reached a consensus on articles to be included in the review. Any disagreements were resolved by consensus with the third author.

### Data extraction

The following data were extracted according to a standard form that included: first author, date, country of publication, study design, sample characteristics and study objectives, mental health assessment, measures of screen time exposure, and main associations found. The allocation of records in the form followed the year of publication, starting with more recent studies.

The term self-reported was used to indicate that screen time was the time spent on screen-based activities, reported by the participant in response to a question. When the study applied a validated instrument or performed an objective measurement of screen time, it was indicated in the form.

### Quality assessment

The methodological quality of the studies was assessed using the Newcastle-Ottawa Scale, based on criteria related to selection and comparability between cohorts and related to study outcomes. The methodological quality of the cross-sectional studies was performed using the adapted Newcastle Ottawa for cross-sectional studies [20]. The evaluation was performed in pairs, by four reviewers and discrepancies were resolved by consensus with a fifth reviewer. The maximum score (9 points) represents high methodological quality [21].

## Results

In the first search carried out on 12/27/2021, 1,309 records were identified in the database through the search strategy. Four hundred and forty-two duplicates were removed and 867 articles were screened by title and abstract. After screening, 763 reports were excluded because they did not meet pre-established criteria. Thus, 104 articles were reviewed, of which 44 were included in this review. These 44 articles were added to the most recent search, carried out on 01/18/2023, totaling 50 articles reviewed in this study. Both selection processes are described in detail in two PRISMA Flowcharts (Additional File 2: Figures 1 and 2).

### Study characteristics

Of the 50 studies, published between 2011 and 2023, 38 were cross-sectional and 12 longitudinal, with a total of 1,900,447 adolescents. Table 2 (Additional file 1: Table 2) presents the distribution of this sample.

Data extracted from the included studies are summarized in Table 1.

**Table 1:** Descriptive characteristics of the included studies

Author, country, type of study	N / Age / Gender / Purposes	Mental Health (MH) – assessment	Screen Time (ST) measurements	Associations
Chen et al. [22] China Cross-Sectional	N: 1,331 M Age: 7–9 th grades 48,69% girls To investigate the associations between different types of (screen-based sedentary behavior - SSB) and anxiety symptoms.	Anxiety symptoms: Zung Self-Rating Anxiety Scale	Self-reported. SSB was categorized into television/movie time, video game time, and internet-surfing time	Video game time of 6 or more hours was positively associated with boys’ anxiety symptoms (p < .05), with a large effect size (OR = 5.12, 95% CI: 1.56–17.44). There were no associations between different types of SSBs and anxiety symptoms in girls.
Forte et al. [23] Ireland Cross-Sectional	N:1,756 M Age: 15,2 56,7% girls To investigate the associations between screen time (ST) and physical activity (PA) level with depressive symptoms.	Depressive Symptoms Quick Inventory of Depressive Symptomatology	Self-reported weekly ST (TV, computer (PC), and phone use)	The most significant correlations with depressive symptoms were shown by higher levels of computer (β = 0.106, p ≤ .000) and phone use (β = 0.138, p ≤ .000), with a small effect size. Only correlations between phone use and depressive symptoms were moderated by PA level.
Kandola et al. [24] UK Longitudinal	N: 4,599 M Age: 14,0 55%girls To investigate how theoretically replacing different screen-uses with exercise might influence future adolescent emotional distress.	The outcome was emotional distress at age 17: Strengths and Difficulties Questionnaire, emotional symptoms subscale	Self-reported. Daily time-use variables were recorded by 24-hour time-use diaries completed over two randomly selected days, one in the week and the other at the weekend.	Substituting 60 min of television or social media use with team sports was associated with a decrement of 0.17 (95%CI, -0.31, -0.04) and 0.15 (95%CI, -0.29, -0.01) in emotional symptom scores, at 17, respectively. Small effect size.
Kjellenberg et al. [25] Sweden Cross-Sectional	N: 1,139 M Age: 13,4 51% girls To investigate the associations between physical activity pattern, sports participation, screen time and mental health.	Anxiety and health-related quality of life (HRQoL): short version of the Spence Children’s Anxiety Scale and Kidscreen-10.	Self-reported: time spent with screens on a weekday and weekend, not including schoolwork.	Only the group of girls who reported ≥ 5 h ST on weekdays or weekends has shown significant associations. These groups had considerably higher anxiety rates compared with those who reported up to 2 h. When controlling for MVPA, this comparison held significant (B = 3.39, 95% CI 1.33 to 5.46). Large effect size.
Kidokoro et al. [26] Japan Cross-Sectional	N: 7,847 M Age: 14,0 53% girls To investigate associations between different types of screen behavior and depression, considering sleep and exercise.	Depression symptoms were measured using a modified version of the depression questionnaire developed by the American Psychiatric Association	Self-reported: time spent on recreational (i.e., screen behavior outside of school) screen, in a week.	Longer time spent on newer sorts of screen behavior, including social media, and online games (among junior high school girls), was associated with a higher prevalence of depression, with a small effect size. Longer time was spent on TV correlation with a lower prevalence of depression
Marciano et al. [27] Switzerland Longitudinal	N: 674 M Age: 14,4 56,7% girls To investigate how changes in screen-media use from Spring 2019 (T1) influenced adolescents’ mental health in (T2) Autumn 2020	Mental health: adapted version of the Diagnostic and Statistical Manual of Mental Disorders, Fifth Edition (DSM-5). Loneliness: 3-item version of the UCLA Loneliness Scale. Depression: seven items from the Center for Epidemiologic Studies Depression Scale.	Self-reported: Screen-media activities were measured at T1 and T2 as time spent in different online activities on “a typical school day” and on “a typical weekend day”	Worse mental health at T2 has only been significantly associated with increased time spent on social media (β = 0.112, p = .016), with medium effect size. Longer time dedicated to structured media activities as television viewing decreased rates of inattention (β = −0.091, p = .021) and anxiety (β = −0.093, p = .014).
Khan et al. [28] 42 European and North American countries. Cross-sectional	N: 577,475 M Age: 13.6 51.35% girls To examine the associations between the combination of ST and Physical Activity (PA) with mental well-being.	Satisfaction with life: Cantril Ladder Psychosomatic complaints: feeling low; irritability or bad mood; nervousness; difficulty falling asleep; dizziness; headaches; stomachaches; and back pain.	Self-reported use of all screen types and all purposes, excluding motion and fitness games.	In a dose-dependent manner, there was negative association between ST levels and life satisfaction and positive association between ST and psychosomatic complaints. Detrimental correlations between ST and mental well-being started when ST exceeded 60 min/d for girls and 75 min for boys (p < .001). Small to medium effect sizes.
McAllister et al. [29] UK Cross-sectional	N: 4,243 M Age: 13.7 55% girls To examine associations between depressive symptoms and self-harm by gender and screen types.	Depression: Short version of the Mood and Feelings Questions (SMFQ); Self-harm: reported whether they had engaged in self-harm in the past year	Self-reported: Time Use Diary (TUD) activities that involved screen media: texting/e-mailing, social media, internet use, gaming and “watching TV, DVDs, downloaded videos” (TV/videos).	More than 3 h/d on social media (n = 92) more likely to self-harm (29%) and less than 1 h/d (n = 852) less likely (19%) girls. For depressive symptoms the results were similar 31% vs. 19%. Small effect sizes. Self-harm or depression among boys was not associated with media use. The correlation was not significant between MH and games or TV/video for boys and girls.
Sampasa-Kanyinga et al. [30] Canada Cross-sectional	N: 6,364 M Age: 15.1 56.7% girls To investigate associations between compliance with the Canadian 24 H Movement Guidelines recommendations and psychological distress.	Psychic distress: 6-item Kessler Psychological Distress Scale (K6)	Self-reported: Time spent in the last 7 days on any screen device in free time.	ST was only associated with sleep duration when combined with anxiety and depression (p < .001), with a small effect size. No direct correlation between ST and anxiety or depression was observed.
Ren et al. [31] China Cross-sectional	N: 1,771 M Age: 12 to 19 51.8% girls To examine the psychological impacts of the pandemic COVID-19 on the development of mood disorders.	Anxiety: General Anxiety Disorder-7 (GAD-7, Chinese version). Depression: Patient Health Questionnaire-9 (PHQ-9, Chinese version); Perceived Social Support: Multidimensional Scale of Perceived Social Support (MSPSS, Chinese version); Psychological resilience: Connor-Davidson Resilience Scale-10 (CD-RISC-10, Chinese version).	Self-reported: average time spent per day, in leisure and study, on electronic devices (TV, smartphones, tablets and computers).	Symptoms of anxiety and depression were 28.3% and 30.8%, respectively. The risk of anxiety/depression decreases with less ST (P < .05). Between ages, the association between TS and depression and anxiety did not differ.
Brown et al. [32] Canada Longitudinal wave 1 (1994–1995) and wave 2 (1996)	N: 6,436 M Age: 16.03 52% girls To determine whether adherence to a movement behavior profile was associated with differences in depressive symptoms.	Depression: Center for Epidemiological Studies Depression Scale (CES-D)	Self-reported: 7-day recall Daily hours spent on TV/videos; computer games.	High profile AF and low ST reported minor depressive symptoms, and these differences were evident one year later
Brown & Kwan [33] Canada Cross-sectional	N: 1,118 M Age: 15.92 54.5% girls To examine the effects of reallocation/substitution of PA, ST, and sleep on mental well-being in adolescents.	Flourishing: Diener Flourishing Scale. Self-esteem: modified Rosenberg Self-Esteem Scale. Resilience: two articles from the Canadian Campus Well-Being Study.	Self-reported: standard 7-day recall. How many hours per day on average are spent watching TV, using the PC, tablet or smartphone during free time.	ST (combined with < 8 h of sleep) is negatively associated with self-esteem, resilience, and flourishing. Replacing 60 min/d of ST with moderate/intense PA or sleep was associated with better self-esteem and resilience scores. For flourishing, benefits were observed when replacing ST with moderate/intense PA or sleep (in the group that slept less than 8 h). Small effect size.
Gilchrist et al. [34] Canada Cross-sectional	N: 46,413 M Age: 9th to 12th grade 51.5% girls To examine the impact on MH when reallocating 15 min spent on one health behavior to 15 min spent on another behavior.	Depression: 10-item Center for Epidemiologic Studies Revised Depression Scale − 10 (CESD-R-10) Anxiety: (GAD-7) Generalized Anxiety Disorder 7-item scale Flourishing: Diener Flourishing Scale.	Self-reported. How much daily time is spent watching TV or movies, playing video/PC games, surfing the Internet, and texting/messaging/e-mailing.	Replacing ST with any behavior (homework, PA, or sleep) may be better for MH outcomes. Small effect size.
Khan & Burton [35] Australia Cross-sectional	N: 2,946 M Age: 16.9 49% girls To investigate associations between two common recreational screen activities and psychological well-being.	Psychological well-being: Strengths and Difficulties Questionnaire (SDQ).	Self-reported: Average daily time spent on electronic games and TV separately.	Playing electronic games was inversely associated with psychological well-being for male and female adolescents (p < .001), with medium and large effects size. Watching TV was inversely associated with psychological well-being for female adolescents (p < .001), with large effect size.
Nigg et al. [36] Germany Longitudinal	N: 686 M Age: wave 1 (T2) 11.85 years wave 2 (T3) 16.86 years 55.2% girls To investigate the relationships between PA, ST and MH.	MH status: Strengths and Difficulties Questionnaire (SDQ), subscales: emotional symptoms; conduct problems; hyperactivity/inattention; relationship problems; pro-social behavior	Self-reported: daily time spent on TV/video, PC/internet and PC games	In girls, TV time at T1 and T2 predicted emotional symptoms at T2 and T3, overall SDQ score and conduct problems, hyperactivity and inattention at T3; PC time at T1 predicted hyperactivity/inattention at T2 and conducted problems at T3; For boys, PC time at T1 predicts emotional symptoms at T2. Conduct problems, hyperactivity/inattention, peer relationship problems, and SDQ score at T2 predicted TV time and PC time at T3.
Twenge & Farley [37] UK Cross-sectional	N: 11,427 M Age: 13.77 50% girls To examine associations between different types of screen activities and MH indicators. And whether there are differences in these associations between genders.	Self-harm behaviors: “In the past year have you purposely hurt yourself in any way?“ with response options of “yes” or “no”; Depression: Short form mood and feelings questionnaire; Self-Esteem: 5 questions based on Rosenberg’s Self-Esteem Scale; Life Satisfaction: 6 questions based on scales that measure life satisfaction.	Self-reported: How much daily time, outside of school, is spent with all types of screen devices (smartphone, tablet, TV, internet, Digital Versatile Disk (DVD), video games, social media)	Among girls, the association of MH problems with social media and Internet use was more evident than for gaming and TV (p < .001). Boys: social media and Internet use are associated with self-harm behavior. Girls: MH impairment when ST ≥ 2 h; for boys, only with > 5 h of ST. In individuals who used social media for more than 5 h, MH problems were more relevant for girls than for boys (p < .05). Small effect size.
Xiao et al. [38] China Cross-sectional	N: 1,680 M Age: 7th to 12th grade 48.7% girls To evaluate PA and ST during the COVID-19 pandemic, and also their associations with mood disorders and conflict with parents.	Mood State: Shortened Chinese version of the Mood States Profile (POMS). Conflict with parents: “Approximately how many times have you had a conflict with your parents in the last 7 days?“	Self-reported: Students reported the number of hours per day they spend studying online and other uses of ST.	More time studying online (p < .05) and other ST (p < .001) verified more conflict with parents. Girls had significantly higher mood disorder scores (p < .01). Other ST use was associated with mood disorders (p < .01). A 1 h addition of ST was associated with a 1.6-to-1.8-point increase in mood.
Bang, et al. [39] Canada Cross-sectional	N: 1,477 M Age: 12 to 17 50.9% girls Correlations to adherence to the 24-Hour Movement and MH Guidelines.	Social difficulties: Strengths and Difficulties Questionnaire (SDQ); Stress: self-reported; MH: self-report	Self-reported: how much time on screen devices.	Adolescents who adhered to ST recommendations were more likely to have good psychosocial health (OR = 2.64; 95% CI: 1.21–5.73) when compared to those who did not (p < .05). Mean effect size.
Barthorpe et al. [40] UK Cross-sectional	N: 4,032 M Age: 13 to 15 55.2% girls To investigate associations between time spent on social media and self-harm, depressive symptoms and self-esteem.	Self-mutilation: yes/no response to the question “In the past year, have you hurt yourself on purpose?“ Depressive symptoms: Short Mood and Feelings Questionnaire. Self-esteem: short Rosenberg scale	ST on social media was recorded in time use diaries (TUD). Social media use was assessed through the activity ‘browsing and updating on social networking sites, e.g., Twitter, Facebook, BBM, Snapchat’.	Significant associations in girls only. Increased time on social media increased the risk of: Automutilation on weekdays (OR = 1.13, 95%CI 1.06 to 1.21). Depression on weekdays (OR = 1.13, 95%CI 1.06 to 1.21). weekend use = 0.19, 95%CI 0.06 to 0.32). Lower levels of self-esteem on weekdays (adjusted OR by 30-min increase in social media use = 1.13, 95%CI 1.06 to 1.21). (adjusted β by 30-min increase in: weekday use = -0.12, 95%CI -0.20 to -0.04; weekend use = -0.12, 95%CI -0.18 to -0.07). Medium effect sizes.
Cao et al. [41] China Cross-sectional	N: 4,178 53.4% girls M Age: 14.2 To identify clustering patterns of health-related behavior and their association with depressive symptoms.	Depressive symptoms: Depression Scale of the Center for Epidemiologic Studies.	Self-reported: daily time spent using all kinds of screen devices.	ST > 2 h/d higher risk of developing depressive symptoms 1,24 (1,06 − 1,45) (p < .01). Cluster of high ST pattern 4.83 ± 1.66 h/d, adolescents were 1.37 times (AOR = 1.37, 95% CI: 1.08–1.73) more likely to develop depressive symptoms compared to Cluster < 2 h/d of ST (p < .01). Small effect size.
Coyne et al. [42] USA Longitudinal	N: 487 M Age: 13 to 20 51.6% girls To assess the correlation between time spent on social media, depression, and anxiety.	Depression: 20-item Depression Scale, Center for Epidemiological Studies (CES-DC). Anxiety: 6-item Spence Child Anxiety Inventory.	Self-reported: Time spent on social networking sites on a typical day.	Girls spend more time on social networking sites. Increased time spent on social networking sites was not associated with increased MH problems when examined for both genders.
Faria et al. [43] Brazil Cross-sectional	N: 217 M Age: 16.08 49.3% girls To identify PA-related lifestyles and sedentary behavior (SB) and their association with health outcomes.	Common Mental Disorders (CMD): General Health Questionnaire, 12-item version, validated in Brazil. Tobacco and alcohol use: short version of the Global School-Based Student Health Survey, validated for Brazilian adolescents.	Self-reported: daily time spent in front of any screen device.	Adolescents included in the “Inactive and Sedentary” class had higher CMD scores than those assigned to the “Active and Non-Sedentary” class (p < .05). Girls with signs of CMD were 9.20 (95% CI 1.17–71.52) more likely to be in the “Inactive and Sedentary” class than in the “Active and Non-Sedentary” class. Medium effect size.
Faulkner et al. [44] Canada Longitudinal	N: 2,292 M Age: 16.3 54% girls To examine whether changes in adherence to the Canadian 24 h Movement Guidelines (PA, ST, and sleep) are associated with changes in flowering.	Flourishing: Diener Flourishing Scale. Depression: Center for Epidemiological Studies Depression Scale-(revised)-10 (CESD-R-10)	Self-reported: Daily time spent watching TV/movies, playing video/PC games, talking on the phone, surfing the Internet, and texting, messaging, or e-mailing.	The majority did not meet the guidelines for total recreation ST (90.5% of the girls and 94.3% of the boys). Meeting the ST guideline was not associated with flowering in both sexes. For girls and boys, meeting more guidelines was not associated with higher flourishing scores when controlling for depressive symptoms and other covariates (p > .05).
Kim et al. [45] Canada Cross-sectional	N: 2,320 M Age: 14.58 50.3% girls Quantify the strength of the association between passive and active forms of ST and adolescent major depressive episodes and anxiety disorders.	Major depressive episode, social phobia, generalized anxiety disorder and specific phobia: Mini International Neuropsychiatric Interview for Children and Adolescents, according to DSM-IV criteria.	Passive ST: Hours /day watch TV, movies, or videos, including YouTube?“ Active screen: hours /day outside of school on average on a PC, laptop, tablet, or smartphone?“	Adolescents with 4 h or more of passive ST p/day, compared to < 2 h, were 3 times more likely to meet DSM-IV-TR criteria for major depressive episode [OR = 3.28 (95% CI = 1.71) -6.28)], social phobia [OR = 3.15 (95% CI = 1.57–6.30)], and generalized anxiety disorder [OR = 2.92 (95% CI = 1.64–5.20)]. Passive ST use was associated with mood and anxiety disorders. Medium effect size.
Weatherson et al. [46] Canada Cross-sectional	N: 29,133 50.26% girls M Age: 15.3 To examine whether complete MH status (CMHS) is associated with adherence to the guidelines for moderate to vigorous PA and recreational ST from the Canadian 24 h Movement Guidelines.	Flourishing: Diener’s Flourishing Scale Depression: Center for Epidemiologic Studies Revised 10-item Depression Scale (CESD-R-10). L: wasting F: flourishing -DS: low depressive symptoms +DS: high depressive symptomatology	Self-reported: daily time watching TV or movies, playing video/PC games, talking on the phone, surfing internet and text messaging, email messaging). To meet the guidelines the sum must be < 2 h p/day of recreational ST.	Presence of DS in 53.85% and F in 50.46%. L/-DS individuals were 50% more likely to meet ST guidelines compared to L/+DS. F/+DS individuals were 87% more likely, and F/-DS were 112% more likely to meet ST guidelines (p < .0001). Lower SD scores and higher F scores were associated with meeting ST guidelines (p < .0001).
Zhang, et al. [47] China Cross-sectional	N: 7,200 M Age: 15.50 50% girls Estimate the combination of exercise time and ST to promote MH. Formulate a benchmark for these variables to prevent the development of psychological problems.	Psychological symptoms: Multidimensional Sub-health Questionnaire of Adolescents (MSQA)	Self-reported: daily time playing video games and watching TV/video programs?“ Every type of ST was counted, from leisure to educational purposes. There were 4 options for teens to choose from: “less than 1 h,“ “1-2 h,“ “2-3 h,“ and “more than 3 h.“	Detection rates for emotional and behavioral symptoms and social adjustment difficulties were higher among boys than among girls. ST > 2 h/d is a risk factor for emotional, behavioral, psychological symptoms, and social adjustment difficulties, with medium effect size.
Orben & Przybylski [48] UK Cross-sectional	N: 355,358 M Age: 16 ± 1.24 50.5% girls Show that specification curve analysis (SCA) can be a more reliable way to show the relationship between well-being and ST.	Revaluation of data obtained from 3 samples of previous studies: Monitoring the Future (MTF); Youth Risk and Behavior Survey (YRBS); Millennium Cohort Study (MCS). Psychological Well-being: Strengths and Difficulties Questionnaire (SDQ); Rosenberg Self-Esteem; Moods and Feelings Scale; 4 items from the Center for Epidemiological Studies Depression Scale	Self-reported: Own computer; Weekday electronic games; Hours of social media use; Weekday TV; Mean technology; Use internet at home	Association between digital technology use and well-being is negative, with a 0.4% of variation in well-being. YRBS: When employing the electronic device, the effects were more negative; when including TV use, however, they were less negative. MTF: Watching TV only on the weekend, median positive association with well-being (β = 0.008); Using social media, median negative association with well-being (β = 0.031).
Khouja et al. [48] UK Longitudinal	N: 14,665 M Age: 16 to 18 51% girls Evaluate the association between ST for device types (watching TV, PC use, and texting), time (weekday or weekend), and anxiety and depression.	Anxiety and depression: computerized version of the Clinical Interview Schedule (CIS-R); Childhood covariates for further adjustment: IQ: Wechsler Intelligence Scale for Children (WISC-III UK); Parental conflict; Father’s presence in the home; Number of people living in the home; Bullying; Early use of family TV.	Self-reported ST: watching TV, PC use and texting. Use during the week and at weekends.	More time spent using PC during the week was associated with a small increase in the risk of anxiety (p = .003). More time spent using the PC only on weekends was associated with a small increase in risk of anxiety and depression (p = .003). There was a small positive association between computer use at age 16 and anxiety and depression two years later.
Liu et al. [49] China Cross-sectional	N: 11,831 M Age: 15,0 50.9% girls To examine the association between the time of cell phone use and depressive symptoms.	Depression: The Center for Epidemiological Studies Depression Scale (CES-D) and the Chinese Youth Self-Report (YSR) of the Achenbach Child Behavior Checklist.	Self-reported: “On an average school day, how many hours have you used a smartphone in the past month?”, “On an average weekend day, how many hours have you used a smartphone in the past month?”	Weekdays: depressive symptoms increased with ST ≥ 2 h/day compared with ST < 1 h/day (19.1% and 10.0% respectively). This association was small. Weekends: depressive symptoms increased with ST ≥ 5 h/day compared with ST < 2 h/day (18.3% and 8.6%, respectively). This association was medium.
Liu et al. [50] China Cross-sectional	N: 13,119 M Age: 15.18 49,5% girls Analyze moderate to vigorous PA (MVPA) and screen-based sedentary behavior (SSB) and their correlations with depression, anxiety, and self-injurious behavior.	Depression: Centers for Epidemiological Studies 20-item Depression Scale Anxiety: 39-item Multidimensional Anxiety Scale Self-injurious behavior: 5-item subscale, Health-Risk Behavior Inventory.	Self-reported. Average daily hours spent watching TV, playing video games, or using the PC on a typical school day. Clusters: 1 (high MVPA/low SSB), 2 (low MVPA/low SSB), 3 (low MVPA/high SSB), 4 (low MVPA/low SSB).	Boys: Depressive symptoms in class 3 > classes 1 and 2 (p < .001). Total anxiety in class 3 > classes 1 (p < .014) and > class 2 (P < .009). Self-injurious behaviors in class 4 > class 2 and class 3 > than classes 1 and 2 (p < .001). Girls: depressive symptoms in class 3 > than classes 1 (p < .001) and 2 (p < .005). Anxiety class 2 > class 1 (p < .042). Both sexes: self-injurious behavior is more severe in class 3.
Paulus et al. [51] USA Cross-sectional	N: 4,257 M Age: 120 months 47.5% girls To investigate associations between Screen Media Activity (SMA) and the psychopathology of internalizing and externalizing symptoms.	Psychological symptoms: Child Behavior Checklist CBCL. Usable structural neuroimaging and SMA data from the Adolescent Brain and Cognitive Development Study (ABCD).	Self-reported: frequency of screen use.	Youth with higher GFA 1 and 4 scores, i.e., had more significant levels of externalizing problems. Also, they had a thinner occipital cortex and a lower volume in the orbitofrontal areas, such as a thinner hippocampus and a lower inferior temporal cortical volume. Individuals with higher SMA-related GFA 1 (β = 0.059) and GFA 4 (β = 0.095) scores had significantly higher externalizing scores, with small effect sizes.
Perrino et al. [52] USA Longitudinal	N: 370 M Age: 14.65 47.6% girls To understand SB based on screen activity and examine the relationship with internalizing psychological symptoms.	Internalizing symptoms: internalizing subscale of the Youth Self-Report. The variable internalizing problems were created by summing three subscales: (a) anxious-depressed b) withdrawn c) somatic complaints	Physical Activity Questionnaire for Adolescents (PAQ-A) SB subscale. Self-report the amount of time spent in the previous week on TV; video games/PC games; texting; email; surfing the Internet and using the telephone.	Girls had more internalizing symptoms and used more internet, email, messaging and cell phones (p < .001). Boys spent more time with video games (p < .001). Positive association between internalizing symptoms and SB in the early phase (p = .01) and their trajectories (p < .001), in both genders (p = .50).
Hrafnkelsdo-ttir et al. [53] Iceland Cross-sectional	N: 244 M Age: 15.8 59% girls To examine separate and interactive associations of ST and physical activity level with MH.	MH problems (depression, anxiety and somatic symptoms): “22-item version of the Subscales of the Symptom Checklist 90”. Self-esteem: Rosenberg Self-Esteem Scale. Life satisfaction: “Diener’s Satisfaction with Life Scale”.	Self-reported: how many h/d on average played PC games, watched TV/DVD/Internet material, used the Internet for web browsing/Facebook/email, and participated in “other” PC use. Participants were classified into high and low ST groups.	Less ST was associated with lower odds of reporting: Symptoms of depression (RR = 0.33, 95% CI = 0.14–0.76) - p < .001. Anxiety (RR = 0.44, 95% CI = 0.23–0.84) - p < .01. Low self-esteem (RR = 0.31, 95% CI = 0.15–0.66) - p < .005. Dissatisfaction with life (RR = 0.38, 95% CI = 0.20–0.72) - p < .005. Associations with small effect size.
Gireesh et al. [54] UK Cross-sectional	N: 120,115 M Age: 15,0 52.42% girls To identify modifiable behavioral factors for mental well-being taking into account deprivation, ethnicity, and grouping.	MH: Warwick-Edinburgh Mental Well-Being Scale (WEMWBS).	Self-reported on weekdays, and weekends, time spent on TV, internet, smartphone and PC games. Categorized into ‘≥7 hours/day’, ‘about 5–6 hours/day’, ‘about 3–4 hours/day’, ‘about 2 hours/day’, and ‘≤1 h/day’.	Being physically inactive, having higher ST, and bullying were associated with decreased well-being in both genders, with the association being more significant in girls (p < .005) than in boys (p < .05). Well-being in both sexes decreased with higher ST in both sexes, starting at about 3 h/d of exposure (p < .001). Associations with small to moderate effect sizes.
Khan et al. [55] Bangladesh Cross-sectional	N: 671 M Age: 14.3 49% girls To explore interactive associations of PA and ST with psychosocial difficulties.	Psychosocial difficulties: Parent-reported Strengths and Difficulties Questionnaire (SDQ)	Self-reported: Adolescent Sedentary Activity Questionnaire (ASAQ) for a typical school day and weekend. Total recreation ST was dichotomized as ≤ 2 h/d (‘low’) or > 2 h/d (‘high’).	Insufficient physical activity + high ST resulted in an 18% increase in total psychosocial difficulties scores, with small effect size. ST was not significantly associated with SDQ difficulties scores (model 2: p = .44).
Twenge et al. [56] USA Longitudinal	N: 506,820 M Age: 13 to 18 50.76% girls To examine cultural changes in 3 generations (GenX, Millennials, and iGen). Determine trends in depressive symptoms, suicide-related outcomes, and suicide rates and examine associations between MH, ST of new and older media, and non-screen activities.	Studies included: Monitoring the Future (MtF) and the Youth Risk Behavior Surveillance System (YRBSS) from 1991 and the Centers for Disease Control data from 1999. Depressive symptoms: MtF: 6 items from the Bentler Inventory of Medical and Psychological Functioning depression scale YRBSS: 4 items related to suicide.	Self-reported. Non-school activities only. 2009: TV, video games, PC games, and internet; 2011: Facebook; 2013 and 2015: smartphone and other social media. Homework. MtF asked, “About how many hours spend on average in a week on all their homework, including both in school and out of school…”	Since 2010, iGen teens have spent more time in new screen media activities and less time in non-screen activities. Teens who spent more time on screen activities were more likely to have high depressive symptoms. Social media use was correlated significantly with depressive symptoms among girls (p < .001). All suicide-related outcomes were significantly with electronic device use (p < .001). Associations with a small effect size.
Yan et al. [57] China Cross-sectional	N: 2,625 M Age: 13 to 18 46.9% girls To determine time spent on screen activities, associations with adiposity, unhealthy eating behaviors, sleep, PA, academic performance, anxiety, self-esteem, and life satisfaction.	Anxiety: High School Student Mental Health Scale. Satisfaction with life: Satisfaction With Life Scale. Self-esteem: Rosenberg Self-esteem Scale.	Self-reported: Time spent watching TV, playing e-games, receiving news or study materials from electronic devices, using social media sites or apps, and watching videos on school and non-school days.	Watching TV during school days for 2 to 4 h was negatively associated with anxiety (p = .047) and self-esteem (p = .032). Receiving news via digital media or studying for 2 to 4 h (p = .036) or > 4 h (p = .002) on school days was positively associated with anxiety. Positive association of social media use with anxiety (p = .009). On school days, watching TV > 04 h is negatively associated with life satisfaction (p = .012), with large to medium effect size.
Khan & Burton [58] Bangladesh Cross-sectional	N: 505 M Age: 14.3 53% girls Assess the association of moderate to vigorous PA (MVPA) with depressive symptoms in adolescents with recreational high ST.	Depression: Center for Epidemiological Studies Depression Scale (CESD 10)	Self-reported: recreational ST (watching TV, DVDs/videos, PC use for entertainment; use of social media - Facebook, Twitter), Adolescent Sedentary Activity Questionnaire (ASAQ).	Adolescents with high recreational ST: a quarter (24.6%) reported depression. A significant amount more girls with high ST than boys reported depression (29% vs. 20%). There were no significant associations between meeting MVPA recommendations and depression for those with low recreational ST (< 2 h/d). Medium effect size.
Przybylski & Weinstein [59] UK Cross-sectional	N: 120,115 M Age: 15,0 Assess how ST is linked to mental well-being and empirically quantify a moderate activity level in digital activities.	Mental well-being: Warwick-Edinburgh Mental Well-Being Scale.	Self-reported: throughout the week, how much of your free time is spent on TV, console, and computer games, internet, email, smartphones, online chats and social networks.	Relationship to impaired mental well-being and watching movies/TV, playing games, and using the PC during the week. Small effect size. For smartphone use, this relationship was observed only on weekdays. Girls spent more time using smartphones, PC and watching videos, and boys spent more time on the PC and console games (p < .001).
Babic et al. [60] Australia Longitudinal	N: 322 M Age: 14.4 66% girls To examine associations between changes in ST (total and device-specific) and MH indicators (well-being and malaise).	Physical self-concept: Physical self-concept subscale from Marsh’s Physical Self-Description Questionnaire. Psychological well-being: Diener and colleagues’ Flourishing Scale. Psychological distress: Strength and Difficulties Questionnaire.	Self-reported: Adolescent Sedentary Activity Questionnaire ASAQ: Recreational time spent using screen devices (TV, PC, tablet/cellphone) each weekday and weekend. Non-recreational ST when a PC was used for homework.	Total recreational ST (p = .048) and tablet/cellphone use (p < .001) were negatively associated with physical self-concept. Total recreational ST (p = .001) and PC use (p = .003) were negatively associated with psychological well-being. There was a positive association between TV/DVD use and psychological difficulties (p = .015). Large effect size.
Goldfield et al. [61] Canada Cross-sectional	N: 358 M Age: 15.6 72.9% girls To examine the association between duration and types of ST and depressive symptomatology in overweight and obese adolescents.	Depressive symptoms: Childhood Depression Inventory (CDI)	Self-reported: How many hours a day spent watching TV, playing video games, and using the computer for recreational purposes. Computer games excluded.	ST is associated with more severe depressive symptomatology (p = .001). Playing video games and recreational PC use was associated with depression (p = .05 and p = .006) respectively, but watching TV showed no significant relationship (p = .09). ST may represent a risk factor for depressive symptomatology. Small effect size.
Gunnell et al. [62] Canada Longitudinal	N: 1,160 M Age: 13.54 60.5% girls To examine changes in PA and ST, depression, and anxiety. Bidirectional relationships between PA, ST, and depression and anxiety.	ST: Self-report questionnaire Depression: Children’s Depression Inventory (CDI). Anxiety: Multidimensional Anxiety Scale for Children-10 (MASC-10).	Self-reported Questions: How many hours per day did the adolescents spend watching TV, playing video games, and using the computer on weekdays and weekends?	Symptoms of depression, anxiety, and ST increased significantly (p < .05) over time. Higher initial symptoms of anxiety are associated with initial symptoms of depression (p < .05) and higher ST (p < .05).
Hayward et al. [63] Australia Cross-sectional	N: 3,295 45.7% girls M Age: 15.14 To establish associations and relative contributions between diet, PA, ST behaviors, and depressive symptomatology.	Depressive symptomatology: Moods and Feelings Questionnaire - Short Form (SMFQ).	ST: Key Indicators and Measures from the Youth Health Survey. Questions: How many hours during the 07 days before the survey did adolescents have ST (less than 01 h, 1-2 h, 2-5 h, and 5 + h).	Achieving the recommended ST guidelines was associated with reduction in the likelihood of depressive symptoms (OR = 0.90 and 95% CI= [0.87, 0.93] in girls. Small effect size.
Trinh et al. [64] Canada Cross-sectional	N: 2,660 52.5% girls M Age: 15.8 To examine the independent effects of PA and ST on MH, school connectedness, and academic performance, and to identify possible interactions between PA and ST in such associations.	Psychological stress: General Health Questionnaire (GHQ) Depressive symptoms: four items adapted from the Center for Epidemiologic Studies Depression (CES-D) Self-esteem: 6 items adapted from the Rosenberg Self-esteem Scale	Self-reported Question: “In the past seven days, how many hours per day did you spend watching TV, playing games, chatting on the computer, or surfing the Internet?“	ST was significantly associated with poor MH outcomes, including psychological distress (p < .05), low self-esteem (p < .05), and depressive symptoms (p < .05). Boys: greater screen time was associated with psychological distress (p < .05), low self-esteem (p < .05), and depressive symptoms (p < .05). Girls: greater ST was associated with low self-esteem (p < .05). Small effect size.
Maras et al. [65] Canada Cross-sectional	N: 2,482 M Age: 14.1 57.8% girls To examine the relationships between sedentary ST and symptoms of depression and anxiety.	Depression: The Children’s Depression Inventory (CDI) (Self-related). Anxiety: Multidimensional Anxiety Scale for Children − 10 (MASC-10)	Leisure-Time Sedentary Activities 6-item questionnaire - measuring how many hours per day students spend on activities such as watching TV, playing video games, and using the PC.	Total ST is associated with depression (p < .001) and severity of anxiety (p < .001). ST related to video games and PC use was associated with more severe depression (p < .001). ST related to video games alone was associated with more severe anxiety (p < .001), with medium effect size.
Suchert et al. [66] Germany Cross-sectional	N: 1,296 M Age: 13.7 47% girls Examine the effects of sedentary behavior (SB) on mental well-being and the differences between screen-based SB (sSB) and non-screen-based SB (nSB).	Depression: German version of the Center for Epidemiological Studies Depression Scale for Children (CES-DC); General self-efficacy: 5 items from Schwarzer and Jerusalem’s general self-efficacy scale; Self-esteem: KINDL-R. Physical attractiveness self-image: physical self-concept scales by Stiller, Würth, and Alfermann.	Self-reported: Time spent on activities involving screens during the last school day and on the last Sunday: watching TV/DVDs, playing video/PC games, and other leisure activities on PC/mobile phone, except active electronic games.	Girls: screen-based sedentary behavior (sSB) is associated with higher depression (p = .032), lower self-esteem (p = .039), lower self-concept for physical attractiveness (p = .003) and lower overall self-efficacy (p = .002). Boys: significant positive association between sSB and self-esteem (p = .014). Small effect sizes.
Nihill et al. [67] Australia Cross-sectional	N: 357 100% girls M Age: 13.2 To examine the association between SB and self-esteem among adolescent girls.	SB: Adolescent Sedentary Activity Questionnaire (ASAQ); Physical self-concept and self-esteem: Marsh’s Physical Self-Description Questionnaire (PSDQ).	ST was created by adding time spent watching TV, videos, DVDs and using the PC for non-school purposes.	Multilevel models did not reveal any association. After corrections, there were inverse associations between time spent watching DVDs (p < .05), playing PC games (p < .05), and total ST (p < .05) and self-esteem. Small effect size.
Straker et al. [68] Australia Longitudinal	N: 643 M Age: 14,0 To examine the relationships between sedentary behavior (including ST) and self-esteem.	MH: Cowen’s Perceived Self-Efficacy Scale. BDI for Youth CBCL - Youth Self-Report version of the Child Behavior Checklist.	Time spent watching TV, playing electronic games, video games and other different PC usages for graphics, text, email, internet and general gaming. Categories: C1 instrumental PC users; C2 multimodal electronic gamers; C3 PC e-gamers.	Instrumental PC users girls had higher self-efficacy than e-gamers girls (p = .028). Instrumental PC boys had lower depression scores than multimodal and e-gamers boys (p = .046), having also less symptoms. Small effect sizes.
Arbour-Nicitopoulos et al. [69] Canada Cross-sectional	N: 2,935 M Age: 15.9 49% girls Investigate the prevalence of psychological distress and its associations with health risk behaviors.	Stress: General Health Questionnaire (GHQ); Substance Use (alcohol, tobacco, and cannabis): Self-report	Self-reported: In the last 7 days, how many hours per day you spend in front of screens.	Students who did not comply with ST recommendations were at higher risk for psychological distress (p < .001). Girls are approximately 2 times more likely than boys to experience psychological distress (p < .001). Tobacco use was significantly associated with the risk of psychological distress (p < .001). Small effect sizes.
Cao et al. [70] China Cross-sectional	N: 5,003 47.9% girls M Age: 13.13 To test the association between ST, PA and psychological problems in adolescents.	Depression: 18-item version of the Self-Regulatory Depression Scale for Children (DSRSC); Anxiety: 41-item Screen for Child Anxiety Related Emotional Disturbances (SCARED); Satisfaction with school life: 12-item School Life Satisfaction Assessment Questionnaire for Adolescents.	Self-reported: How much time, on average, I spend on weekdays and weekends on sedentary activities, such as watching TV and using a PC.	Positive associations of ST with depression, anxiety, and dissatisfaction with school life (p < .001). High ST was a risk factor for: Depression (OR = 1.52–95%CI: 1.31–1.76); Anxiety (OR = 1.36, 95%CI : 1.18–1.57); Dissatisfaction with school life (OR = 2.07, 95% CI : 1.79–2.38). Small effect sizes.

### Participants characteristics

Participants’ ages ranged from 10 to 21 years, mean 14,85 standard deviation 1,14. Some studies presented data on the age of participants based on school grade [22, 34, 38]. In the pooled sample, there was a greater participation of girls, however, one study did not provide this data [59], and there was a study that evaluated only girls [67]. Of the studies that reported sample demographic data, the socioeconomic status was mostly medium and predominantly white ethnicity.

### Screen time and mental health assessments

The measurements taken for both screen time and mental health were very heterogeneous. Most of the time, screen time was self-reported and aspects such as flourishing and resilience were considered in the mental health assessment. The data are described in detail in (Additional file 3).

### Associations between screen time and mental health

Most studies have observed associations between screen time and adolescent mental health. Only a few studies found no unfavorable associations between screen time and overall mental health, or any of its aspects [22, 26, 27, 29, 38, 42, 48, 56, 57, 60, 66, 67]. For the studies that found significant associations, most of the time, the findings were from just one survey, or congruent among a few studies, which did not allow performing a statistical analysis of the associations. A small number of studies showed an effect size from medium to large [35, 57, 65] or large [22, 25, 35, 60]. Most made sizes were small [23, 26, 29, 30, 33, 34, 37, 41, 51, 53, 55, 56, 59, 61, 63, 64, 66, 68–71], or medium [27, 28, 39, 40, 43, 45, 47–49, 57, 58].

### Cross-sectional associations

#### Types, contents, and usage habits of screens

According to a study, with a robust sample of adolescents in the United Kingdom, the screen activities with the highest levels of engagement were social media, games and TV/video [29]. Watching TV was positively associated with mood and anxiety disorders [45], impaired mental well-being [59], i.e., the more TV time, the higher scores for mood and anxiety disorders and greater impairment in mental well-being, and inversely associated with self-esteem and life satisfaction [57] and psychological well-being for girls [35]. Whereas in other studies, TV was negatively associated with anxiety [57], positively with mental well-being [48] and lower prevalence of depression [26]. Furthermore, one study [61] did not find associations between watching TV and depressive symptoms, and another [29] also found no associations with mental health.

While Kim et al. (2020) [45] did not find associations of active screen use with mood and anxiety disorders, and McAllister et al. (2021) [29] between mental health and gaming, other studies did. A relationship was observed between computer use (such as for internet, email, and games) with impairment of mental well-being [59], psychological well-being [35] and an increase in depressive symptoms [61, 65]. Video games alone were associated with more severe anxiety symptoms [65]. And 6 h or more of video gaming was positively associated with anxiety symptoms in boys [22]. Higher levels of computer use showed stronger association with depressive symptoms [23]. And online gaming (among high school girls) was associated with a higher prevalence of depression [26]. The studies do not specify the modality online or not of video gaming.

The smartphone was the device that adolescents report more time using, according to Przybylski & Weinstein (2017) [59]. A recent study observed that telephone use showed a stronger association with depressive symptoms among the girls [23], while in another study it represented impairment of mental well-being only on weekdays [59]. More time spent on new types of screen behavior, including social media, was associated with a higher prevalence of depression in one study [26]. Social media use also had a median negative association with well-being in another study [48]. Among girls, the positive association of mental health problems with social media and internet use was greater than for games and TV in the study by Twenge et al. (2021) [37]. In McAllister et al. (2021) [29], media use negatively impacted mental health, but was not significantly associated with self-harm or depression among boys.

Among adolescents with high recreational ST, about a quarter reported depressive symptoms in one study [58]. Girls who reported ≥ 5 h of ST on weekdays or weekends had higher anxiety scores compared to those who reported up to 2 h, even controlling for moderate to vigorous physical activity (MVPA), in a recent survey [25]. Time studying online was positively associated with anxiety in one study [57], but was not associated with mood disturbances in another [38], and in the latter, other ST uses were associated with mood disturbance [38].

#### Different mental health outcomes including well-being

Young people who met screen time recommendations were about 2.6 times more likely to have good psychosocial health outcomes compared to those who did not in one study [39]. Higher flourishing scores were associated with meeting ST guidelines of less than 2 h daily in one study [46]. One study found that high ST (with < 8 h of sleep) was negatively associated with self-esteem, resilience, and flourishing [33].

The study by Gireesh et al. (2018) [34] also addressed well-being, finding greater screen time, and suffering from bullying associated with decreased well-being in both sexes, with the strongest association in girls. Playing electronic games was inversely associated with psychological well-being for adolescents of both sexes. Watching television was also inversely associated with psychological well-being in girls in one study [35].

The association between the use of digital technology and adolescent well-being is negative, but small, representing less than 0.1% of the observed variability in well-being, according to the study by Orben and Przybylski (2019) [48]. Watching TV only on the weekend showed a median positive association with well-being. Social media use had a median negative association with well-being [48]. In a previous study, Przybylski et al. (2017) [59], observed a relationship with impaired mental well-being and watching movies/TV, playing games and using the computer throughout the week. As for smartphone use, this relationship was observed only on weekdays.

### Longitudinal associations

#### Types, contents, and usage habits of screens

Social media use was significantly correlated with depressive symptoms among girls but not among boys, moreover, all suicide-related outcomes were correlated with electronic device use in Twenge et al. (2018) [56]. In the study by Coyne et al. (2020) [42], the increase in time spent on social media was not associated with an increase in mental health problems, when adolescents were examined at the individual level. In a recent study, more time spent in structured media activities, such as watching television, decreased levels of inattention and anxiety [27].

An 11-year study found that increased TV viewing and Personal Computer (PC) use was predictive of conduct problems, hyperactivity, and inattention in girls [36]. There was a small positive association between computer use at age 16 and anxiety and depression two years later in one study [71]. Boy computer instrumental users had lower depression scores and fewer internalizing behavior problems than “e-gamers” in one study [68].

#### Different mental health outcomes including well-being

In the study by Babic et al. (2017) [60], decrease in total recreational screen ST was negatively associated with physical self-concept and psychological well-being, and there was a positive association between television/DVD use and psychological difficulties. CP time positively predicted emotional symptoms in one study [36]. Girl instrumental computer users had higher self-efficacy compared to female computer e-gamers in one study [68].

In a recent study, increased time spent on social media was the only screen media activity significantly associated with worse mental health [27]. In another more recent study, the theoretical replacement of 60 min of television or social media use by team sports at age 14 years was associated with a reduction in emotional symptom scores at age 17 years, respectively [24].

### Quality assessment of studies

The summary of the methodological evaluation of the articles included in this review are presented in Table 4, according to the items of the Newcastle-Ottawa scale for observational studies, (Additional file 4: Table 4). The quality assessment of the cross-sectional studies was carried out by adapting the Newcastle Ottawa for cross-sectional studies. Most studies had high methodological quality, with a total score above 6. Recurrent problems among studies were the lack of a group not exposed to screens, generating comparability only between factors such as gender, in addition to the lack of objective measurement of screen time.

## Discussion

Although screen-based activities bring many benefits, such as communication and entertainment, most of the results of this study indicate that excessive exposure to screens is associated with effects on the mental condition of adolescents. Of the 50 studies reviewed, only 12 found no unfavorable associations between screen time and overall mental health, or any of its aspects [22, 26, 27, 29, 38, 42, 48, 56, 57, 60, 66, 67]. It is also important to consider that some studies in this review were carried out during the COVID-19 pandemic and with social distancing, screen time may not significantly negatively interfere with well-being, since it is the only way to if you remain socially connected [10].

### Screens and mental health: do device and content matter?

The current review is consistent with other reviews that concluded that the type, use and content of the screen influences the relationship between mental health problems and ST [11, 13, 72]. It seems that the impairment of mental health in adolescents is closely related to the purpose of screen use and not just the exposure time. For example, online study or non-recreational use of screens [38, 60] does not seem associated with mental health. Currently, there is a suggestion that the term “screen time” is no longer a useful construct [73], since the devices and the social character of the media must be evaluated separately, the nature of the content may be more relevant for mental health than the amount of time teenagers are exposed to screens [74–76]. In fact, in this review, watching TV did not show a significant relationship with depressive symptoms [26, 61], self-esteem [67] or mental health [29], in some cross-sectional studies, it even decreased levels of inattention and anxiety longitudinally [27]. It was also observed in the literature that, over time, the relationship between screen time and depressive symptoms varied between different screen uses, with stronger relationships observed with cell phones and computer/internet, new forms of technology [11].

A very popular activity among teenagers is the use of social media such as Facebook, Instagram, and Twitter [77]. Here, social media use was associated with poor mental health and impaired mental well-being [24, 26, 27, 37, 48, 59]. Our results involving social media agree with previously published reviews [15, 18]. In fact, excessive use of social media can lead to the development of fear of missing out (FoMO). FoMO is defined by the fear that other people will have pleasurable experiences while the individual is away, felt as a need for constant contact with members of the social network [78]. It is true that in adolescence internalizing symptoms happens frequently [79] and here these symptoms were the most common. Thus, the association between social media use and internalizing symptoms may be complex. Social media can worsen depression and anxiety [80], however, adolescents having depression and anxiety symptoms may lean on technology to alleviate those feelings too. In this sense, our results and other revised longitudinal data agreed, that a stronger relationship was observed between greater exposure to screens and a subsequent increase in the depression score [11].

Regarding games, the results were mixed, in McAllister et al. (2021) [29] associations with mental health were not significant for boys and girls. Boers et al. (2019) [76] also did not find a significant association between the time spent playing video games with depression, these authors consider that video game players are not socially isolated, they play with friends, physically or online, which it has social and emotional benefits [81]. However, few studies had already observed a significant association between playing video games and worse mental health in adolescents [82, 83].

In this review, non-recreational computer use for girls was associated with greater self-efficacy, while boys had lower depression scores and internalizing behaviors than those who used their computers only as a game console (“e-gamers”) [68]. The authors suggest that adolescents were acquiring more computer skills, which had already been associated with improved mental well-being in a previous study [84]. In this sense, our data are confirmed in studies over time in which depression did not increase at the intrapersonal level [75, 76].

Smartphone and social media use has been associated with depression [23, 24, 26, 27] and internalizing symptoms [52]. Twenge in 2017 [85] already raised the concern on whether smartphones were “destroying a generation”. Odgers (2018) [86], however, concluded that this would be a misinterpretation of reality, as most adolescents are doing well in the digital age and that US and European numbers show academic improvement, a decrease in violence, abuse of alcohol, smoking, and teenage pregnancy [3, 86]. Smartphone studies can have limitations, such as an often underestimate smartphone use, leading to low correlations between self-reported screen time data and data collected through the device app itself [87, 88]. Considering screen time simply by counting frequency and duration may limit understanding of an adolescent’s relationship with the smartphone and the consequences on mental well-being [89]. It would be important to obtain detailed information about the goals and how the adolescent uses their device [90].

#### Screens and mental health: mediators and confounding factors

In one study, significant associations between anxiety and depression appeared when screen time was combined with shorter sleep duration [30]. It is not yet established whether the act of looking at the screen interrupts sleep or whether the media content is responsible. Light-emitting diode (LED) screens on computers and phones emit a slow wave, blue light, which can interfere with the circadian rhythms that regulate sleep. Exposure to LED versus non-LED screens produces changes in melatonin levels and sleep quality, and this exposure decreases cognitive performance [91]. Sleep disturbances may also be related to excessive use of technological devices at night [92]. Sleep disorders is an umbrella term, according to the International Classification of Diseases 11th Revision (ICD-11), they belong to an overlapping area between mental health and neurological disorders, and according to WHO they are part of common mental health disorders [93–95]. Sleep disorders are often associated with depression and anxiety and often co-occur [94, 96, 97] or even antecede the disorders diagnosis. Currently, young people interact on social networks, sending messages and selfies, sometimes all night, a characteristic behavior that gave rise to the term “Vamping”. This term relates to tech-addicted teens who already have a disrupted circadian rhythm and who are at greater risk of declining school performance and loss of self-control [98]. In fact, in another study of this review [33], self-esteem, resilience and flourishing were negatively associated with ST in those adolescents who slept less than eight hours a night.

In addition to sleep, physical activity may also protect against the potentially harmful effect of interacting with social media in some adolescents [80]. From the records reviewed, some studies noted that replacing screen time with physical activity showed a positive effect on associations with mental health [24, 33, 34]. Insufficient physical activity and high ST was associated with increased psychosocial difficulties [55]. In fact, a review that investigated moderating variables of associations between ST and depression in youth found that physical activity can influence the magnitude of these associations [72].

Increased screen time was significantly associated with aspects of mental health. Few studies showed an effect size from medium to large [35, 57, 65] or large (21,24,34,60). However, most studies observe small effect sizes [23, 26, 29, 30, 33, 34, 37, 41, 51, 53, 55, 56, 59, 61, 63, 64, 66, 68–71], or medium [27, 28, 39, 40, 43, 45, 47–49, 57, 58]. Our findings are agreed with previous literature about the predominant small effect size, in addition to the large heterogeneity of the studies [9, 99].

The results of this review suggest that interaction with screen-based devices may underlie the impairment of adolescent mental health over the last decade. However, other studies also agree that establishing causality and directionality can be difficult [17, 48, 73, 90]. While research is still being conducted, care must be taken in interpreting data on the positive and negative effects of adolescents’ interaction with digital technologies. However, even with possible benefits, it may not be healthy to suppress the other activities that our nature is qualified for, underutilizing our other senses, and looking at screens for most of the day.

## Conclusion

This study contributes with data on the various mental health outcomes of adolescents, including aspects of positive mental health, in addition to considering exposure to all types of screens most used by this population. This review found some evidence for the current research question, we highlight here that watching TV for 2 to 4 h on school days was negatively associated with anxiety and self-esteem. The most time spent by adolescents was with the smartphone and use during the week was associated with diminished mental well-being. Screen exposure time was most positively associated with problems in teens’ mental well-being. Social media use had a median negative association with mental well-being in adolescents and an increased risk of depression in girls. Furthermore, “screen time” may no longer be appropriate for investigations of the effects of exposure to screen-based devices and related mental health outcomes in adolescents. Most of the reviewed studies provided total measures of time spent in front of screens, however, the nature of the content offered on each device, as well as the interaction of adolescents with this content, is still unclear.

More detailed studies will be needed, seeking to understand the motivations of adolescents to engage with screen devices. Studies that consider issues related to the environment of adolescents may also help to clarify the varied emotional responses to screen stimuli. Longitudinal studies that pay attention to factors such as sleep, physical activity and socioeconomic status will also be important to establish mediators of associations between interaction with screens and mental health in this population.

### Limitations

The current review has some limitations, which may impact the generalizability of the results. The first refers to the diversity and fragility of the methods applied in the data collection of the included studies. Self-reported screen time may provide inaccurate data due to adolescents’ recall difficulties. Studies focusing on sedentary behavior that included screen time in this category may provide even coarser measures of this variable. The lack of measurement of adolescents’ interaction time with each type of device and type of content leads to a superficial assessment of the associations between screen time and adolescents’ mental well-being, especially considering the emotional particularities of this phase. The wide variety of instruments to assess mental health outcomes can also be a factor that makes it difficult to standardize results. The second limitation is related to studies that analyzed data from previous research. These studies, in addition to addressing many variables not reviewed in the present study, used old data with a reality different from that observed today. In addition, of the articles reviewed, only one study from Brazil and five from Australia represented the global south, it is necessary to increase diversity. And finally, as adolescents’ interactions with screens and their contents can vary depending on when they occur and for what purpose the screens are being used, the associations between screen time and mental health depend on intensity and context, such as the day of screen use in a week, for the purpose of use and whether the use is recreational or for study.

## Electronic supplementary material

Below is the link to the electronic supplementary material.

Supplementary Material 1

Supplementary Material 2

Supplementary Material 3

Supplementary Material 4

## Data Availability

All data generated or analyzed during this study are included in this published article.

## Abbreviations

AAP: American Academy of Pediatrics
ABCD: Adolescent Brain and Cognitive Development
ADHD: Attention Deficit Hyperactivity Disorder
AOR: Adjusted Odds Ratio
ASAQ: Adolescents’ Sedentary Activities Questionnaire
BBM: Blackberry Messenger
CBCL: Child Behavior Checklist
CD-RISC-10: Connor-Davidson Resilience Scale-10
CDI: Childhood Depression Inventory
CES-D: Center for Epidemiological Studies Depression Scale
CESD-R-10: 10-Item Center for Epidemiologic Studies Depression Scale Revised-10
CES-DC: Children of the Center for Epidemiological Studies
CI: Confidence interval
CMD: Common Mental Disorders
CP: Conduct Problems
DS: Depressive Symptoms
DSM IV: Diagnostic and Statistical Manual of Mental Disorders - Fourth Edition
DSRSC: Self-Regulatory Depression Scale for Children
DVD: Digital Versatile Disk
FoMO: Fear of Missing Out
GAD-7: General Anxiety Disorder-7
GFA: Group Factor Analysis
GHQ: General Health Questionnaire
IQ: Intelligent Quotient
K6: 6-item Kessler Psychological Distress Scale
LED: Light-Emitting Diode
MARCA: Multimedia Activity Recall for Children and Adolescents
MASC-10: Multidimensional Anxiety Scale for Children
MCS: Millennium Cohort Study
M: Mean
MH: Mental Health
MVPA: Moderate to Vigorous Physical Activity
N: Sample
NSB: Non-Screen-Based
PA: Physical Activity
PAQ-A: Adolescent Physical Activity Questionnaire - Sedentary Behavior Subscale
PC: Personal Computer
PHQ-9: Patient Health Questionnaire-9
PICO: Population, Intervention, Comparation and Outcome
POMS: Mood States Profile
PRISMA: Preferred Reporting Items for Systematic Reviews and Meta-Analyses
SAMHSA: Substance Abuse and Mental Health Services Administration
SB: Sedentary Behavior
SCA: Specification Curve Analysis
SCARED: Screen for Child Anxiety Related Emotional Disturbances
SDQ: Strengths and Difficulties Questionnaires
SMA: Screen Media Activity
SMFQ: Short Form of the Mood and Feelings Questionnaire
SSB: Screen-Based Sedentary Behavior
ST: Screen Time
TV: Television
TUD: Time Use Diary
UCLA: (University of California, Los Angeles) Loneliness Scale
UFMG: Universidade Federal de Minas Gerais (Federal University of Minas Gerais)
USA: United States of America
UK: United Kingdom
WEMWBS: Warwick-Edinburgh Mental Well-Being Scale
WHO: World Health Organization
WISC-III UK: Wechsler Intelligence Scale for Children
YRBSS: Youth Risk Behavior Surveillance System
YRSB: Youth Risk and Behavior Survey
YSR: Youth Self-Report
