# Supplementary material for: The associations between screen time and mental health in adolescents: a systematic review
Source: BMC Psychol. 2023 Apr 20;11:127. doi: 10.1186/s40359-023-01166-7 (PMC10117262; doi:10.1186/s40359-023-01166-7)
Supplement: Supplementary file 1 — Supplementary Material 1 [file 40359_2023_1166_MOESM1_ESM.docx]

Additional file 2: Figure 1- First Search

Total studies included in review

(n = 50)

New studies included in review

(n = 6)

Records screened

(n = 208)

Reports sought for retrieval

(n=20)

Reports assessed for eligibility

(n = 17)

**Identification**

Records identified through database searching
(n = 1309)

Records duplicates
(n =442)

Records after duplicates removed
(n =867)

**Screening**

Records excluded
(n =763)

Records screened
(n =867)

Full-text articles excluded, with reasons (n =60)

- Out of age = 25

- Didn’t measured mental health with validated scale = 17

- Didn’t associated directly screen time and mental health= 14

- Sample with problematic screen usage =2

- Screen used as an aid = 1

- Sample with mental health problem = 1

Full-text articles assessed for eligibility
(n = 104)

**Eligibility**

Additional records identified through full-text reading
(n = 0)

(n = 1)

**Included**

Studies included in qualitative synthesis
(n = 44)

PRISMA Flow Diagram

Additional File 2: Figure 2- Updated Search

**Previous studies**

Records identified from:

Databases (n=307)

Records removed *before screening*:

Duplicate records removed

(n=99)

Records screened

(n = 208)

Records excluded by a human

(n=187)

Reports sought for retrieval

(n=20)

Reports not retrieved

(n =3)

Reports assessed for eligibility

(n = 17)

Reports excluded: *

Reason 1 (n =2)

Reason 2 (n =5)

Reason 3 (n =2)

Reason 4 (n =1)

Reason 5 (n =1)

New studies included in review

(n = 6)

**Identification of new studies via databases and registers**

**Identification**

**Screening**

**Included**

Total studies included in review

(n = 50)

Studies included in previous version of review (n =44)

* Reason 1: Did not use validated scale to assess mental health

*Reason 2: Mix children in the adolescent sample

*Reason 3: Outside the scope of the study

*Reason 4: Did not directly associate ST and MH

*Reason 5: Sample with problematic screen usage
