# Supplementary material for: The associations between screen time and mental health in adolescents: a systematic review
Source: BMC Psychol. 2023 Apr 20;11:127. doi: 10.1186/s40359-023-01166-7 (PMC10117262; doi:10.1186/s40359-023-01166-7)
Supplement: Supplementary file 2 — Supplementary Material 2 [file 40359_2023_1166_MOESM2_ESM.docx]

**The associations between screen time and mental health in adolescents: A Systematic Review**

Additional file 3: Assessments

**Screen Time Assessment**

Screen time (ST) was self-reported and established in different ways by the studies in this review. The study by [44] compared passive and active screen use. Some studies (n=14) have considered screen time as sedentary behavior. Seven studies verified compliance or not with the 24-hour movement guidelines established in Canada, that consist on a composite analysis including 9 to 11 hours of sleep per night for 5 to 13 years old and 8 to 10 hours per night for 14 to 17 years old, 60 minutes of moderate to vigorous physical activity and less than 2 h/day of screen time. One study separately quantified time watching television and playing video games[34]. Another study excluded computer games [35]. Studies considered only recreational screens (n=5), the recreational screen on different devices was accounted separately [36], or just the time spent on social media[41]. But most articles (n=36) considered screen time as covering online studies and all other uses.

Different patterns of screen time evaluation were observed. One study measured screen time using the Multimedia Activity Recall for Children and Adolescents (MARCA) [68]. Some studies applied t sedentary activities questionnaire (ASAQ), considering screen-based recreational activities [67,55,58] and school activities [60]. One study used the Leisure-Time Sedentary Activities questionnaire (46) and the other the Adolescent Physical Activity Questionnaire - Sedentary Behavior Subscale (PAQ-A) [52]. The Time Use Diary (TUD) was used in three studies, for all screen uses [23], for the use of social networks only [39] and separately for digital media (games) and social networks [28]. Some studies used the strategy of creating clusters, classes or behavior profiles, such as physically active or sedentary individuals. These studies created lifestyle-based outcome blocks that included physical activity and screen time [42, 50], sleep, physical activity, and screen time [31, 45], and another that stratified into profiles of different types of screen-based devices [68].

**Mental Health Assessment**

Other outcomes were evaluated in addition to mental health aspects, such as school performance or even physical performance; however, we focused on the mental health-evaluation. Of the 50 articles reviewed, the symptoms evaluated were depression (n=10), depressive symptoms (n=22), anxiety (n=16), and self-esteem (n=9). Some studies focused on positive aspects of mental health, highlighting mental well-being, and evaluating dimensions such as adolescent flourishing (n=5) and resilience (n=1).

Some studies evaluated dimensions that reflect the general state of mental health, such as life satisfaction (n=5), self-efficacy (n=2), physical self-concept (n=3), social difficulties (n=3), psychosocial difficulties (n=1) and stress (n=2). In addition to emotional symptoms, some studies have evaluated behavioral symptoms, including conduct problems, hyperactivity/inattention, and pro-social behavior (n=4). Engagement in self-harm behavior was addressed in some articles (n=6) and suicidal ideation was assessed in two studies [47, 56]. One study evaluated mental disorders defined by the criteria of the Diagnostic and Statistical Manual of Mental Disorder (DSM IV), for a major depressive episode, generalized anxiety disorder, social phobia, and specific phobia [44]. One study assessed overall mental health using the adapted version of the Diagnostic and Statistical Manual of Mental Disorders, Fifth Edition (DSM-5) and also assessed loneliness using the (University of California, Los Angeles) Loneliness Scale- UCLA [26]. Studies that referred to mood disorders [30,37, 44] evaluated anxiety and depression. Two studies assessed psychological distress [64,69], and one emotional distress (Kandola et al., 2022). Two studies evaluated internalizing and externalizing symptoms [51,68] and one study (47) evaluated only depression as an internalizing symptom. Exposure to alcohol and tobacco, as a determinant of mental health, was also addressed in a study [42]. On the other hand, some studies have focused their efforts on capturing positive aspects of adolescent mental health, such as positive mental health [43], psychological Well-Being (38), Well-Being [32,54,59] .

Due to the geographic and methodological diversity of the included studies, 42 scales used to assess the mental health of adolescents were identified. The most used scales were the 10-item Center for Epidemiologic Studies Depression Scale Revised−10 (CESD-R-10), Rosenberg Self-Esteem Scale, Strengths and Difficulties Questionnaire (SDQ), Short Form of the Mood and Feelings Questionnaire (SMFQ) and the Diener's Flourishing Scale.
