# Supplementary material for: The associations between screen time and mental health in adolescents: a systematic review
Source: BMC Psychol. 2023 Apr 20;11:127. doi: 10.1186/s40359-023-01166-7 (PMC10117262; doi:10.1186/s40359-023-01166-7)
Supplement: Supplementary file 3 — Supplementary Material 3 [file 40359_2023_1166_MOESM3_ESM.docx]

Additional file 1

Table 1. Search strategies

| **Data Base** | **Search strategies** |
| --- | --- |
| Pubmed | All fields (((adolescent) AND (screen time)) AND (mental health) |
| PsycInfo | All Fields: adolescent AND All Fields: screen time AND All Fields: mental health |
| Scopus | All Fields: adolescent AND All Fields: screen time AND All Fields: mental health |

Additional file 1

Table 2. Distribution of samples in the included studies

| Country | Number of studies | Total participants |
| --- | --- | --- |
| Canada | 13 | 105.148 |
| China | 9 | 47.451 |
| United Kingdom (UK) | 7 | 634.554 |
| Australia | 5 | 7.563 |
| United States of America (USA) | 4 | 511.954 |
| Germany | 2 | 1.982 |
| Bangladesh | 2 | 1.176 |
| South Korea | 1 | 54.243 |
| Iceland | 2 | 2.000 |
| Japan | 1 | 7.847 |
| Sweden | 1 | 1.139 |
| Switzerland | 1 | 674 |
| Brazil | 1 | 217 |
| 42 North American and European countries | 1 | 577.475 |
