# Supplementary material for: The associations between screen time and mental health in adolescents: a systematic review
Source: BMC Psychol. 2023 Apr 20;11:127. doi: 10.1186/s40359-023-01166-7 (PMC10117262; doi:10.1186/s40359-023-01166-7)
Supplement: Supplementary file 4 — Supplementary Material 4 [file 40359_2023_1166_MOESM4_ESM.docx]

**The associations between screen time and mental health in adolescents: A Systematic Review**

Additional file 4: **Quality Assessment**

Table 4 - Quality assessment of studies - Newcastle-Ottawa Scale

| **References** | **Design** | **Selection** | **Compatibility** | **Outcome** | **Total** |
| --- | --- | --- | --- | --- | --- |
| Chen et al. [21] | Cross-sectional | 3 | 2 | 2 | 7 |
| Forte et al. [22] | Cross-sectional | 3 | 2 | 3 | 8 |
| Kandola et al. [23] | Prospective Cohort | 2 | 2 | 2 | 6 |
| Kjellenberg et al. [24] | Cross-sectional | 3 | 2 | 3 | 8 |
| Kidokoro et al. [25] | Cross-sectional | 3 | 2 | 2 | 7 |
| Marciano et al. [26] | Prospective Cohort | 2 | 2 | 2 | 6 |
| Khan et al. [27] | Cross-sectional | 3 | 2 | 3 | 8 |
| McAllister et al. [28] | Cross-sectional | 3 | 2 | 3 | 8 |
| Sampasa-Kanyinga et al. [29] | Cross-sectional | 3 | 2 | 3 | 8 |
| Ren et al. [30] | Cross-sectional | 3 | 2 | 3 | 8 |
| Brown et al. [31] | Prospective Cohort | 2 | 2 | 2 | 6 |
| Brown et al. [32] | Cross-sectional | 3 | 2 | 3 | 8 |
| Gilchrist et al. [33] | Cross-sectional | 3 | 2 | 3 | 8 |
| Khan et al. [34] | Cross-sectional | 3 | 2 | 3 | 8 |
| Nigg et al. [35] | Prospective Cohort | 2 | 2 | 2 | 6 |
| Twenge et al. [36] | Cross-sectional | 3 | 2 | 3 | 8 |
| Xiao et al. [37] | Cross-sectional | 2 | 2 | 3 | 7 |
| Bang et al. [38] | Cross-sectional | 3 | 2 | 2 | 7 |
| Barthorpe et al. [39] | Cross-sectional | 3 | 2 | 3 | 8 |
| Cao et al. [40] | Cross-sectional | 3 | 2 | 3 | 8 |
| Coyne et al. [41] | Prospective Cohort | 2 | 2 | 2 | 6 |
| Faria et al. [42] | Cross-sectional | 2 | 2 | 3 | 7 |
| Faulkner et al. [43] | Prospective Cohort | 2 | 2 | 3 | 7 |
| Kim et al. [44] | Cross-sectional | 3 | 2 | 3 | 8 |
| Weatherson et al. [45] | Cross-sectional | 4 | 2 | 3 | 9 |
| Zhang et al. [46] | Cross-sectional | 3 | 2 | 3 | 8 |
| Orben et al. [47] | Cross-sectional | 3 | 2 | 3 | 8 |
| Khouja et al. [48] | Prospective Cohort | 3 | 2 | 3 | 8 |
| Liu et al. [49] | Cross-sectional | 3 | 2 | 3 | 8 |
| Liu et al. [50] | Cross-sectional | 3 | 2 | 3 | 8 |
| Paulus et al. [51] | Cross-sectional | 3 | 2 | 3 | 8 |
| Perrino et al. [52] | Prospective Cohort | 2 | 2 | 3 | 7 |
| Hrafnkelsdottir et al. [53] | Cross-sectional | 2 | 2 | 3 | 7 |
| Gireesh et al. [54] | Cross-sectional | 3 | 2 | 3 | 8 |
| Khan et al. [55] | Cross-sectional | 2 | 2 | 3 | 7 |
| Twenge et al. [56] | Prospective Cohort | 3 | 2 | 3 | 8 |
| Yan et al. [57] | Cross-sectional | 3 | 2 | 3 | 8 |
| Khan et al. [58] | Cross-sectional | 2 | 2 | 3 | 7 |
| Przybylski et al. [59] | Cross-sectional | 3 | 2 | 3 | 8 |
| Babic et al. [60] | Prospective Cohort | 1 | 2 | 3 | 6 |
| Goldfield et al. [61] | Cross-sectional | 2 | 2 | 3 | 7 |
| Gunnell et al. [62] | Prospective Cohort | 3 | 2 | 3 | 8 |
| Hayward et al. [63] | Cross-sectional | 3 | 2 | 3 | 8 |
| Trinh et al. [64] | Cross-sectional | 3 | 2 | 3 | 8 |
| Maras et al. [65] | Cross-sectional | 2 | 2 | 3 | 7 |
| Suchert et al. [66] | Cross-sectional | 3 | 2 | 3 | 8 |
| Nihill et al. [67] | Cross-sectional | 3 | 2 | 3 | 8 |
| Straker et al. [68] | Prospective Cohort | 2 | 2 | 3 | 7 |
| Arbour-Nicitopoulos et al. [69] | Cross-sectional | 3 | 2 | 3 | 8 |
| Cao et al. [70] | Cross-sectional | 3 | 2 | 3 | 8 |

Strong evidence: high quality studies 6/9; Moderate evidence: low quality studies and/or one high quality study 4-5/9; Limited evidence: lower quality study < 4.
